# Relationship between Endothelial Function, Antiretroviral Treatment and Cardiovascular Risk Factors in HIV Patients of African Descent in South Africa: A Cross-Sectional Study

**DOI:** 10.3390/jcm10030392

**Published:** 2021-01-20

**Authors:** Benedicta N. Nkeh-Chungag, Nandu Goswami, Godwill A. Engwa, Constance R. Sewani-Rusike, Vuyolwethu Mbombela, Ingrid Webster, Patrick De Boever, Harald H. Kessler, Evelyn Stelzl, Hans Strijdom

**Affiliations:** 1 Department of Biological and Environmental Sciences, Faculty of Natural Sciences, Walter Sisulu University PBX1, 5117 Mthatha, South Africa; bnkehchungag@wsu.ac.za (B.N.N.-C.); gengwa@wsu.ac.za (G.A.E.); 2 Physiology Division, Otto Loewi Research Center for Vascular Biology, Immunology and Inflammation, Medical University of Graz, Neue Stiftingtalstrasse 6, D-5 A 8036 Graz, Austria; 3 Department of Human Biology, Faculty of Health Sciences, Walter Sisulu University PBX1, 5117 Mthatha, South Africa; crusike@wsu.ac.za (C.R.S.-R.); vuyombo@gmail.com (V.M.); 4 Centre for Cardio-Metabolic Research in Africa, Division of Medical Physiology, Faculty of Medicine and Health Sciences, Stellenbosch University, PO Box 241, 8000 Cape Town, South Africa; iwebster@sun.ac.za (I.W.); jgstr@sun.ac.za (H.S.); 5 Department of Biology, University of Antwerp, 2610 Wilrijk, Belgium; Patrick.DeBoever@uantwerpen.be; 6 Centre for Environmental Sciences, Hasselt University, 3590 Diepenbeek, Belgium; 7 Health Unit, Flemish Institute for Technological Research (VITO), 2400 Mol, Belgium; 8 Diagnostic & Research Institute of Hygiene, Microbiology and Environmental Medicine, Medical University of Graz, Neue Stiftingtalstrasse 6, AT-8010 Graz, Austria; harald.kessler@medunigraz.at (H.H.K.); evelyn.stelzl@medunigraz.at (E.S.)

**Keywords:** human immunodeficiency virus, antiretroviral treatment, cardiovascular disease, endothelial function, obesity, hypertension

## Abstract

Limited information on the effect of antiretroviral treatment (ART) on vascular function in South Africans of African descent living with human immunodeficiency virus (HIV) is available. The relationship between ART, vascular function and cardiovascular risk factors in South Africans of African ancestry with HIV was therefore studied. This cross-sectional study recruited 146 HIV-positive individuals on ART (HIV^+^ART^+^), 163 HIV-positive individuals not on ART (HIV^+^ART^−^) and 171 individuals without HIV (HIV^−^) in Mthatha, Eastern Cape Province of South Africa. Flow-mediated dilation (FMD) test was performed to assess endothelial function. Anthropometry and blood pressure parameters were measured. Lipid profile, glycaemic indices, serum creatinine as well as CD4 count and viral load were assayed in blood. Urinary albumin to creatinine ratio (ACR) was determined as a marker of cardiovascular risk. Obesity and albuminuria were positively associated with HIV, and HIV^+^ART^+^ participants had significantly higher HDL cholesterol. Dyslipidaemia markers were significantly higher in hypertensive HIV^+^ART^+^ participants compared with the controls (HIV^+^ART^−^ and HIV^−^ participants). FMD was not different between HIV^+^ART^+^ participants and the controls. Moreover, HIV^+^ART^+^ participants with higher FMD showed lower total cholesterol and LDL cholesterol comparable to that of HIV^−^ and HIV^+^ART^−^ participants. A positive relationship between FMD and CD4 count was observed in HIV^+^ART^+^ participants. In conclusion, antiretroviral treatment was associated with cardiovascular risk factors, particularly dyslipidaemia, in hypertensive South Africans of African ancestry with HIV. Although, ART was not associated with endothelial dysfunction, flow-mediated dilatation was positively associated with CD4 count in HIV-positive participants on ART.

## 1. Introduction

Global reports show that approximately 17.9 million deaths recorded globally are due to cardiovascular diseases (CVDs) [1]. Notably, 75% of these deaths occur in low- and middle-income countries [1]. Additionally, approximately 1 million deaths were attributed to CVDs in sub-Saharan Africa alone in 2013, which constituted 5.5% of all global CVD-related deaths. The burden of CVDs has been projected to double in sub-Saharan Africa by 2030 [2], In South Africa, CVDs are estimated to account for 18% mortality [3], which is linked to the increased prevalence of cardiovascular risk factors, particularly obesity and hypertension [4,5]. People living with HIV (PLHIV) may be at increased risk of stroke, myocardial infarction, atherosclerosis and chronic kidney disease development as a result of increased traditional cardiovascular risk factors [6,7]. A recent meta-analysis and systematic review reported that the crude rate of CVDs in PLHIV was 61.8 per 10,000 person-years, suggesting that PLHIV were twice as likely to develop CVDs within sub-Saharan Africa, presenting the most disability-adjusted life year (DALY) loss of 0.87 million [8]. The prevalence of cardiovascular risk factors in HIV continues to rise creating a major public health concern in South Africa [9].

Cardiovascular risk in HIV is a complex interplay of several factors. Studies show that obesity and dyslipidaemia are prevalent in HIV patients [10]. Hypertension is becoming a growing problem in adults with HIV. A 6-year prospective cohort study with over 80,000 HIV-infected persons showed that HIV-infected adults with hypertension had a 2-fold higher risk of developing acute myocardial infarction than uninfected adults with hypertension [11]. While traditional risk factors such as obesity, dyslipidaemia, hyperglycaemia and hypertension are known causes of CVD, recent evidence suggests that HIV-associated inflammation, oxidative stress and endothelial dysfunction are important mediators [12,13].

Endothelial dysfunction, resulting in altered vasodilation, endothelium-mediated vasomotor and anti-inflammatory responses [14], is an initiator and early marker for atherosclerosis [15]. Endothelial (dys)function can be assessed by flow-mediated dilation (FMD) of the brachial artery [16]. Although endothelial dysfunction has been associated with CVDs [17], the relationship of HIV infection with endothelial dysfunction needs further clarification [18]. Studies postulated that pro-inflammatory cytokines and immune mediators in HIV may induce endothelial cell injury [10] or HIV-1 virus and its component proteins may directly injure the endothelium resulting in endothelial dysfunction [19]. Furthermore, it is proposed that HIV-induced dyslipidaemia and metabolic syndrome may cause endothelial dysfunction [20].

The introduction and increased use of antiretroviral treatment (ART) has greatly reduced the global burden of HIV/AIDS. There has been a dramatic decline in immunodeficiency-related events, including deaths, in HIV-infected individuals [21,22]. In South Africa, there are over 7.2 million PLHIV and more than 50% of them are on ART medication [23]. Although the use of ART has substantially increased the life expectancy in South Africa, several reports have documented increased prevalence of cardiovascular risk factors, such as obesity, elevated blood sugar, elevated blood pressure, hypertriglyceridaemia and low high-density lipoprotein cholesterol (HDL-c) in PLHIV on ART [24]. ART has also been reported to promote platelet reactivity, a condition which has been implicated in the pathogenesis of atherothrombosis and CVDs [25]. Moreover, there is growing concern that the metabolic complications associated with HIV and ART may lead to an increased risk for CVDs [26].

There is also paucity of information on the effects of ART on vascular function in sub-Saharan Africa [27]. Though South Africa has the greatest global burden of HIV, there is limited information on the effect of ART on vascular function in South Africans of African ancestry who have the greatest burden of the disease in the country. Therefore, the aim of the current study was to specifically assess the relationship between ART, endothelial function and cardiovascular risk factors in a South African population of African ancestry.

## 2. Materials and Methods

### 2.1. Study Design and Population

This was a cross-sectional study that involved adult (≥18 years) South Africans of African ancestry with/without HIV attending the Gateway Clinic, a primary healthcare centre in Mthatha, Eastern Cape Province. Study participants were randomly recruited between June 2016 and December 2019. Participants who were HIV-positive (HIV^+^) and currently on ART medication (HIV^+^ART^+^) were classified separately from participants who were newly diagnosed with HIV and not yet on ART (HIV^+^ART^−^), while those who were HIV-negative (HIV^−^) served as control. Participants considered HIV^+^ART^+^ are those who had received ART for ≥4 months. Participants were tested and their HIV status was confirmed before allocation to any of the study groups, and appropriate pre- and post-test counselling was performed. This study was part of the EndoAfrica South African Project [28]. Based on the protocol of the EndoAfrica study [28], this study had sufficient power to demonstrate 1–2% effect size in FMD endothelial function measurement (80% statistical power and 5% significance) when including 450 participants, of which 150 were HIV^−^ and 300 were HIV^+^ (HIV^+^ART^−^ and HIV^+^ART^+^).

### 2.2. Inclusion/Exclusion Criteria

Male or female adults above 18 years old of African ancestry who had fasted, were either HIV positive or negative at the time of recruitment and were attending the Gateway Clinic in Mthatha without any severe illness or complications were recruited for the study. Participants who were critically ill with or without fever, pregnant, lactating, severely anaemic, physically challenged especially in the right arm used for flow mediated dilation test, had not fasted and individuals of non-African ancestry were excluded from the study. Participants who had smoked or exercised in the past 4 h were also excluded from the study.

### 2.3. Ethical Approval

This study was conducted in accordance with the principles stated in the Declaration of Helsinki (reviewed version of 2008) as well as local and national regulations in South Africa. Ethical approval was obtained from the Health Sciences Ethics Committee of Walter Sisulu University, South Africa (Ref No:005/2015). After careful explanation of the study aims and methods, written informed consent was obtained from participants before recruitment into the study. The study adhered to the standards of reporting and in accordance with the National Data Protection Act, wherein each participant was assigned a code and data/samples were stored anonymously.

### 2.4. Data Collection

A validated interviewer-administered questionnaire was adopted from the WHO STEPwise approach to Surveillance (STEPS) guidelines for non-communicable diseases (NCDs) [29]. It was modified accordingly to fit the aim of the study to obtain information on demographics, lifestyle factors such as smoking and alcohol consumption, physical activity, diet, information on HIV status, ART administration and family history of cardiovascular disease (CVD) risk factor assessment.

### 2.5. Anthropometric Measurements

Anthropometric measurements were performed in accordance with the International Standards for Anthropometric Assessments [30] on all participants. Waist circumference (WC) and hip circumference (HC) were measured using an anthropometric tape in centimetres (cm) and waist-to-hip ratio (WHR) was calculated. Height was measured using a wall-mounted Harpenden stadiometer (version 602, SERITEX) and recorded to the nearest 0.1 cm. Weight was measured using an Omron bioimpedance scale (OMRON BR511) and the body mass index (BMI) was calculated. The WHO classification for obesity by WHR in adults [31] was used to classify obesity as WHR ≥0.90 cm for men or ≥0.85 cm for women.

### 2.6. Blood Pressure Measurements 

Blood pressure was measured in a quiet room using the Omron automated sphygmomanometer (HBP-1100; Omron Healthcare Co. Ltd., Kyoto, Japan). After resting for 5 min, arm-size-appropriate cuffs were fitted to the upper arm and systolic blood pressure (SBP), diastolic blood pressure (DBP) and heart rate (HR) were measured trice automatically at 2 min intervals and the average of the three readings was computed. Hypertension was classified as SBP ≥ 140 mmHg and/or DBP ≥ 90 mmHg [32].

### 2.7. Blood Collection and Biochemical Analysis 

Fasting blood and urine samples were collected. Serum was obtained after centrifugation and the fasting glucose (FG), glycated haemoglobin (HbA1c), triglycerides (TG), total cholesterol (TC), low-density lipoprotein cholesterol (LDL-c), high-density lipoprotein cholesterol (HDL-c), blood creatinine, CD4 count and HIV viral load were determined. Urine creatinine and albumin were quantified from early morning urine samples and the albumin-to-creatinine ratio (ACR) was calculated. The FG, HBA1c, CD4 count, TC, TG, LDL-c, creatinine and urea were determined using a cobas 501/502 panel/system (Roche Diagnostics, Indianapolis, IN, USA) according to the manufacturer’s protocol, while HDL-c was calculated using the Friedewald equation. The cobas 6800/8800 HIV-1 test (Roche Diagnostic, USA) was used to determine HIV viral load by quantifying RNA copies according to the manufacturer’s protocol. All assays were performed in the clinical pathology, haematology and virology units of the National Health Laboratory Service (NHLS) of the Nelson Mandela Academic Hospital, Walter Sisulu University, Mthatha, South Africa. Lipid profile measures were classified as high when TC ≥ 6.2 mmol/L, LDL-c ≥ 4.15 mmol/L and TG ≥ 1.7; and low when HDL-c ≤ 0.90 mmol/L according to the Adult Treatment Panel (ATP) III guideline [33]. Albuminuria (AU) was considered as ACR > 3 mg/mmol (equivalent to ACR > 30mg/g) according to the National Institute for Health and Clinical Excellence (NICE) guideline [34].

### 2.8. Assessment of Vascular Function 

A mobile Esaote MyLab Five portable ultrasound device (Genoa, Italy) with an Esaote Doppler probe (LA523, 12 MHz) connected to computerized software with edge detection technology (Cardiovascular Suite (CVS); Quipu, Pisa, Italy) was used to measure endothelium-dependent flow-mediated dilation (FMD) of the brachial artery. Participants who had fasted overnight and had not smoked or exercised for at least 4 h were asked to lie down on the exam bed in the supine position with their right arm placed on the probe holder of the CVS ultrasound. A blood pressure cuff was placed around the forearm, as close as possible to the elbow and participants rested for 5–10 min prior to FMD measurement. An electrostatic gel probe was used to scan through the upper right arm in a transverse plain to visualize the pulsating brachial artery. Once the brachial artery was visualized on the CVS ultrasound screen, the probe was fixed to the probe holder and maintained in place for the rest of the measurement. The CVS software was turned on for the baseline recording at rest which lasted for 60 s (1 min), after which the artery was occluded by raising the blood pressure to 200 mmHg and maintaining it at this level for 5 min. At the end of the 5 min, the cuff was deflated to allow blood to flow freely in the brachial artery for 2 min after which the recording was stopped. The software then automatically calculated baseline diameter (the diameter of the artery at rest), maximum diameter (the diameter of the artery at occlusion), recovery diameter, baseline shear rate, maximum shear rate and percentage FMD. FMD (%) was calculated as the difference between maximum and baseline diameter, expressed as a percentage of baseline diameter. FMD (%) was classified into interquartile ranges to assess cardiovascular risk factors exceeding critical threshold for endothelial function. Concordance in the operation of the device by operators was checked to ensure reproducibility of the technique.

### 2.9. Data and Statistical Analysis

Statistical Package for Social Sciences (SPSS) version 23.0 (IBM Corp., Armonk, NY, USA) was used for data analysis. Descriptive statistics was employed and data were presented as mean ± standard deviation (SD). Analysis of variance (ANOVA) was used to compare mean differences between HIV^−^, HIV^+^ART^−^ and HIV^+^ART^+^ participants. Multivariate analysis of variance (MANOVA) was done to assess the interaction of antiretroviral treatment with endothelial function, hypertension or obesity among study groups. The relationship of HIV and ART with cardiovascular risk factors was assessed using the Chi-square test of association. Pearson’s correlation was used to assess relationship between endothelial function and CVD risk factors. The standardized beta coefficient (β) was determined by stepwise linear multiple regression analysis adjusted for age, sex and smoking status to identify factors that are predictors of endothelial (dys)function. An alpha error (α) of 0.05 was taken and *p*-value ≤ 0.05 was considered to be significant.

## 3. Results

### 3.1. Characteristics of Study Population

The baseline characteristics of the study population are shown in Table 1. A total of 841 individuals were approached for the study and only 480 individuals, who were eligible, consented to participate in the study. Among the 480 (238 females and 242 males) participants recruited for the study, 171 were HIV^−^ participants, 146 were HIV^+^ participants on ART (HIV^+^ART^+^) and 163 were HIV^+^ participants not on ART (HIV^+^ART^−^). Among the 146 HIV^+^ART^+^ participants, 120 (82.2%) were on TEE (tenofovir disoproxil fumarate + emtricitabine + efavirenz), 11 (7.5%) on TLD (tenofovir disoproxil fumarate + lamivudine + dolutegravir), 7 (4.8%) on zidovudine + lamivudine + efavirenz, 3 (2.1%) on stavudine + lamivudine + nevirapine, 3 (2.1%) on zidovudine + didanosine + lopinavir/ritonavir and 2 (1.4%) on zidovudine + lamivudine + lopinavir/ritonavir. The age and heights of the participants were different (*p* < 0.05) among the three study groups. The HR of HIV^+^ART^+^ participants was significantly (*p* < 0.001) lower than that of HIV^+^ART^−^ and HIV^−^ participants. HDL-c level was significantly (*p* < 0.01) increased in HIV^+^ART^+^ participants compared with HIV^+^ART^−^ participants. The CD4 count was significantly (*p* < 0.001) lower in HIV^+^ART^−^ and HIV^+^ART^+^ participants than in HIV^−^ subjects. The HIV viral load was significantly (*p* < 0.01) lower in HIV^+^ART^+^ participants than in HIV^+^ART^−^ participants and 23.3% of the HIV^+^ART^+^ participants had undetectable viral load levels.

### 3.2. Relationship of HIV and ART with Cardiovascular Risk Factors

The relationship of HIV and ART with cardiovascular risk factors is shown in Table 2. Seventy-one participants (14.8%) in the study population were hypertensive, while 35.4% (170/480) participants in the study population were obese. Among the participants, more HIV^+^ART^−^ participants were obese compared with HIV^−^ and HIV^+^ART^+^ participants and thus, obesity was associated with HIV (χ2 = 18.8; *p*-value = <0.001). Fewer HIV^+^ART^+^ participants had low HDL-c compared with HIV^−^ and HIV^+^ART^−^ participants and thus, ART was associated with increased HDL-c (χ^2^=14.74; *p* = 0.022). More HIV^+^ participants (*n* = 16) had albuminuria compared with HIV^−^ participants (*n* = 1) and thus, albuminuria was associated with HIV status (χ^2^ =7.707, *p* = 0.021).

### 3.3. The Interaction between Hypertension and ART with Cardiovascular Risk Factors

To assess the contribution of hypertension and ART on cardiovascular risk in participants, a two-way analysis of variance (ANOVA) was performed. A significantly increased TC (F_(2,96)_ = 5.349; *p* = 0.006) and LDL-c (F_(2,96)_ = 5.675; *p* = 0.005) level was observed in hypertensive HIV^+^ART^+^ participants compared with the controls. The concentration of TG was also significantly higher (F_(2,96)_ = 4.455; *p* = 0.014) in hypertensive HIV^+^ART^+^ participants and hypertensive HIV^−^ participants. These findings indicated that ART administration was associated with lipid abnormalities in hypertensive HIV participants on ART (Table 3).

### 3.4. Relationship of Endothelial Function and ART With Cardiovascular Risk Factors in HIV 

FMD was classified into interquartile ranges to assess endothelial function in relationship with ART and cardiovascular risk factors. HIV^+^ART^+^ participants with low FMD (second quartile) showed a significantly *(p* < 0.05) higher level of TC and LDL-c compared with HIV^+^ART^−^ and HIV^−^ participants. Moreover, HIV^+^ART^+^ participants with higher FMD (third and fourth quartiles) showed lower TC and LDL-c comparable to that of HIV^−^ and HIV^+^ART^−^ participants. This implied that improved endothelial function was associated with reduced TC and LDL-c in HIV participants on ART (Table 4).

### 3.5. Relationship between ART, Endothelial Function and Cardiovascular Risk Factors

**Figure 1:**
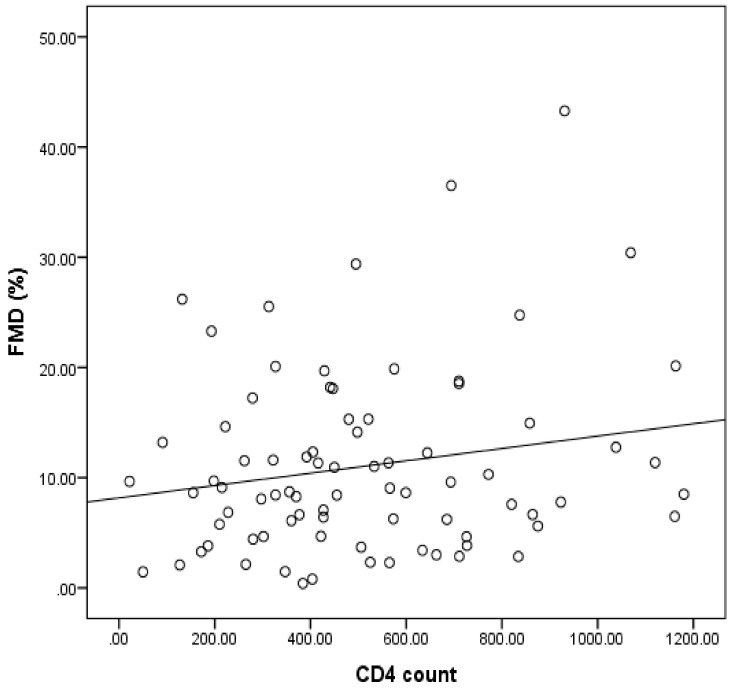
Relationship between FMD (%) and CD4 count on HIV participants on ART. A significant positive correlation (*p* < 0.05) between FMD (%) and CD4 count was observed in HIV participants on ART.

FMD was not associated with viral load (*r* = −0.149; *p* = 0.16) or cardiovascular risk factors (TC, TG, HDL-c, LDL-c, ACR, SBP, DSP and HR) in the overall cohort and various study groups. In addition, there was no correlation between FMD and CD4 count in HIV^+^ART^−^ participants (*r* = −0.072; *p*-value = 0.579) or in HIV^−^ participants (*r* = 0.062; *p*-value = 0.807). However, a significant positive correlation (*r* = 0.250; *p* = 0.023) was observed between FMD and CD4 count in HIV^+^ART^+^ participants (Figure 1). Multiple linear regression analysis adjusted for age, sex and smoking showed no association of FMD with HIV viral load as well as cardiovascular risk factors. Moreover, adjusted stepwise linear regression analysis for a fitted model (*F* = 8.202; *p* = 0.012) showed CD4 count as a positive predictor for FMD (*R^2^* = 0.354; adjusted *R^2^* = 0.31; β = 0. 595; *p* = 0.023) in HIV^+^ART^+^ participants, indicating that ART increases CD4 count which may improve endothelial function in HIV-infected individuals.

## 4. Discussion

In this study, antiretroviral treatment was associated with cardiovascular risk factors, particularly dyslipidaemia, in hypertensive South Africans of African ancestry living with HIV. Previous studies have shown PLHIV to be at increased risk of CVDs [35,36]. Treatment with ART has also shown a positive association with cardiovascular risk factors in some studies [37,38], although findings from other studies have not been conclusive [39,40]. Some studies have shown that ART medication may impair vascular function in individuals with HIV in Western and Caucasian populations [41,42], but similar studies in African populations are not available. The lack of data on the subject in South Africans of African ancestry who present high prevalence of HIV prompted this study to investigate the relationship between HIV infection, ART and cardiovascular risk factors. This study showed no association between obesity and ART (Table 2). This finding is in contrast to previous studies [36,37]. However, a recent study in South Africa showed that the likelihood of an HIV patient on ART being obese was dependent on a longer duration of the treatment [43] and the type of ART administered as some ART do not promote lipogenesis. Therefore, the lack of association between ART and obesity in this study might have been affected by the duration of ART use and the type of ART administered. Obesity was positively associated with HIV in ART-naive patients (Table 2). Factors other than ART, such as smoking and alcohol, may be responsible for this association as has been reported in a previous study [44]. However, this finding is in agreement with some previous studies, which showed that cardiovascular risk factors, including obesity, are positively associated with HIV, although the association is usually not higher than that observed in individuals without HIV [45].

There have been reports that suggest ART as a major contributor to cardiovascular risk in PLHIV [46,47]. Certain ARTs have been shown to promote lipid abnormalities which are associated with CVDs [48,49]. Studies have shown that lopinavir/ritonavir (LPV/r) and fosamprenavir/ritonavir (FPV/r) are associated with increased plasma lipid levels [50]. Certain nucleoside/nucleotide reverse transcriptase inhibitors (NRTIs), including stavudine, abacavir and zidovudine, have been associated with dyslipidaemia, while tenofovir disoproxil fumarate (TDF) or tenofovir alafenamide fumarate (TAF) has been shown to favour lipogenesis [51]. Efavirenz, which belongs to the non-NRTIs, has been associated with increased triglyceride and total cholesterol levels [52]. Findings in this present study showed increased TC, LDL-c and TG levels in hypertensive HIV^+^ART^+^ participants compared with the controls (HIV^+^ART^−^ and HIV^−^ participants), suggesting a positive association between ART and dyslipidaemia in hypertensive PLHIV on ART (Table 3). This finding suggested that ART may increase lipids and cholesterol as previously reported [48], which may in turn promote a pro-atherosclerotic process and arterial stiffness, leading to an increase in blood pressure. Our findings are in agreement with previous studies, which have shown that some ART drug classes, particularly the NRTIs such as zidovudine and TDF, are associated with lipid abnormalities [49,51]. However, it remains unclear why such an association was observed mostly in hypertensive HIV participants on ART. The lack of association between ART and obesity in this study may be a result of the low use of dolutegravir (DTG)-based combination regiments, especially with TAF, which has been shown to be associated with weight gain and clinical obesity [53]. However, HDL-c was higher in HIV^+^ART^+^ participants compared with HIV^+^ART^−^ individuals (Table 1). This suggested that ART may favourably improve HDL-c levels in circulation. Considering the importance of HDL in the removal of cholesterol from circulation [54], ART in this study may have beneficial effects in cholesterol management due to the increased HDL-c level. A few studies have confirmed an elevated HDL-c level in HIV^+^ART^+^ individuals, suggesting a protective role [55,56], while other studies have shown ART to reduce HDL-c in HIV-infected patients [24,57] as opposed to the findings obtained in this study.

Albuminuria is another independent cardiovascular risk factor and has been associated with CVDs. Indeed, ACR, a measure for albuminuria, is a marker of renal function and cardiovascular risk [58]. A 10-fold as well as a 5-fold increase in urine albumin level, characteristic of albuminuria, was observed in HIV^+^ART^+^ and HIV+ART^−^ participants, respectively, compared with HIV^−^ participants. Moreover, there was a trend of reduced ACR and albumin in HIV^+^ART^+^ participants compared with their HIV^+^ART^−^ counterparts (Table 1). The proportion of albuminuria in HIV^+^ individuals on/not on ART was also higher compared with HIV^−^ individuals, translating to an association between HIV infection and albuminuria (Table 2). This finding suggested that albuminuria, which was associated with HIV, may be improved by ART as previously reported [59]. This finding agrees with a few studies which have shown the proportion of albuminuria to be high in persons with HIV [60,61].

Endothelial dysfunction is one of the key steps that contributes to the initiation and clinical manifestations of atherosclerosis and known to be an early predictor of future cardiovascular events in patients with or without a known cardiovascular risk [16,62]. Some studies have shown associations between HIV infection and endothelial dysfunction [10] and impaired endothelial function has previously been shown in HIV-infected individuals on ART [63] as well as in obese animals treated with antiretroviral drugs [64]. However, this finding remains controversial as some studies have not been able to establish an association between ART and endothelial dysfunction [65]. The findings in this study did not suggest that HIV or ART might affect endothelial function as flow mediated-dilation was not different between HIV^+^ART^+^, HIV^+^ART^−^ and HIV^−^ participants (Table 1). The underlying reason explaining this observation is not known. We speculate that duration of ART treatment, types of ART regimens and ethnicity may play a role. In this context, we provided one of the first observations for an African population and follow-up studies should further challenge our findings. This study also showed that, although TC and LDL-c were higher in HIV^+^ART^+^ participants with low FMD (second quartile) compared with HIV^+^ART^−^ and HIV^−^ participants, HIV^+^ART^+^ participants with higher FMD (third and four quartiles) showed lower TC and LDL-c comparable to that of HIV^−^ and HIV^+^ART^−^ participants (Table 4). This indicated that reduced endothelial function may be associated with lipid abnormality, while improved endothelial function may reduce lipid abnormality in HIV participants. There was also a positive relationship between FMD and CD4 count in HIV^+^ART^+^ participants (Figure 1) and CD4 count showed approximately a 0.6 chance (β = 0.595) to improve endothelial function, that is, increased CD4 count was independently associated with improved endothelial function. This finding suggested that ART may improve CD4 count, which can ameliorate endothelial dysfunction in HIV subjects. A possible explanation for this observation is that ART may help to reduce HIV viral load (evident by the increased CD4 count), which has been suggested to cause endothelial damage [19]. Therefore, reduced HIV viral load in tissues, which is accompanied by increased CD4 count, may reduce endothelial damage.

Several influencing participant-related covariates and environmental factors could lead to low reproducibility of FMD measurements [66], but strict adherence to operating guidelines has been shown to provide reliable FMD measurements [67,68,69]. Therefore, in this study, we ensured reproducible measurements and a low coefficient of variation for FMD by executing the standard operating procedures described in the aforementioned papers as well as by Strijdom and colleagues [28]. Aspects such as limiting the number of operators, extensive training of the operator, measuring the same brachial artery regions, analysis in a controlled environment at approximately the same time of the day and regular quality control of the data were observed. The study employed three operators for FMD measurements and they were checked to ensure the reproducibility of results. Though adherence to strict operating guidelines was applied, this study may have been limited by the fact that treatments that could affect FMD, such as anti-aggregants, anticoagulants or statins, were not accounted for. There was also a disparity in the ages of participants in the various study groups and as a cross-sectional study, conclusions on causality cannot be drawn. This study could not correct for the different ART regimens, as they differ in effect on cholesterol and other cardiovascular risk factors which could affect the overall findings. We performed multiple statistical testing to discover biological trends and thus, we increased the risk of false positives, and follow-up studies will need to confirm our findings.

## 5. Conclusions

Antiretroviral treatment was associated with cardiovascular risk factors, particularly dyslipidaemia, in hypertensive South Africans of African ancestry living with HIV, but it was not associated with endothelial function. Moreover, antiretroviral treatment was associated with increased HDL-c in HIV participants. Although ART was not associated with endothelial function, flow-mediated dilatation was positively associated with CD4 count in HIV-positive participants on ART. Considering that this was a cross-sectional study which may be limiting, there is a need for longitudinal studies in the future to further assess the relationship of ART with cardiovascular risk factors in this population.

## Figures and Tables

**Table 1:** Baseline characteristics of study population.

	HIV^−^	HIV^+^ART^−^	HIV^+^ART^+^	*p*-Value
N	171	163	146	
Smoking (P/A)	42/128	33/127	28/118	0.295
Diabetes (P/A)	1/170	1/162	1/145	0.993
Age (years)	28.9 ± 7.9	31.9 ± 6.9	34.4 ± 8.4	<0.001
Weight (kg)	70.1 ± 15.9	66.9 ± 14.9	70.8 ± 16.8	0.077
Height (cm)	165.9 ± 7.9	163.13 ± 7.3	163.8 ± 9.2	0.006
HC (cm)	103.3 ± 13.2	104.4 ± 14.7	100.6 ± 15.2	0.093
WC (cm)	85.2 ± 15.7	88.9 ± 16.2	88.4 ± 16.9	0.091
BMI (kg/m^2^)	25.5 ± 6.1	25.1 ± 6.2	26.5 ± 6.9	0.136
WHC	0.9 ± 0.5	0.9 ± 0.1	0.9 ± 0.1	0.917
SBP (mmHg)	124.6 ± 18.1	123.0 ± 34.9	121.5 ± 25.9	0.528
DBP (mmHg)	81.7 ± 12.5	78.0 ± 17.9	79.9 ± 19.3	0.132
HR (bpm)	77.3 ± 25.8	80.04 ± 18.8	65.9 ± 23.4 ^a,b^	<0.001
TC (mmol/L)	3.9 ± 0.9	3.9 ± 0.9	4.6 ± 5.1	0.114
HDL-c (mmol/L)	1.3 ± 0.4	1.2 ± 0.4	1.4 ± 0.5 ^b^	0.004
LDL-c (mmol/L)	2.1 ± 0.9	2.2 ± 0.8	2.6 ± 0.7	0.557
TG (mmol/L)	1.1 ± 0.6	1.1 ± 0.6	1.3 ± 0.8	0.058
FG (mmol/L)	4.9 ± 0.6	4.9 ± 1.1	5.3 ± 0.9	0.127
HBA1c (%)	5.4 ± 0.4	5.4 ± 0.5	5.4 ± 0.4	0.738
BC (μmol/L)	70.2 ± 13.9	66.0 ± 14.2	67.6 ± 13.8	0.072
UC (mmol/L)	14.2 ± 7.9	14.5 ± 8.2	12.6 ± 5.8	0.163
Albumin (mg/L)	2.8 ± 8.3	22.4 ± 38.2	11.1 ± 18.5	0.011
ACR (mg/mol)	0.2 ± 0.7	1.2 ± 5.0	0.9 ± 1.8	0.062
eGFR ((mL/min)	140.8 ± 56.9	136.9 ± 47.6	129.1 ± 41.6	0.273
CD4 count (cells/μL)	941.2 ± 1123.7	425.0 ± 328.7 ^a^	519.9 ± 273.8 ^a^	<0.001
Viral load (1000 copies/mL)	-----	171.5 ± 319	27.8 ± 132	0.003
FMD (%)	12.5 ± 10.1	14.5 ± 10.4	12.2 ± 11.8	0.162

Analysis of variance was used for analysis. Results are expressed as mean ± standard deviation (SD). HIV: human immunodeficiency virus, HIV^+^: HIV-positive subjects, HIV^−^: HIV-negative subjects, ART: antiretroviral treatment, HIV^+^ART^−^: HIV-positive participants not on ART, HIV^+^ART^+^: HIV-positive participants on ART, ^a^ indicates significant difference with HIV^−^ subjects, ^b^ indicates significant difference with HIV^+^ART^−^ participants. P: present, A: absent, HC: hip circumference, WC: waist circumference, BMI: body mass index, WHR: waist-to-hip ratio, SBP: systolic blood pressure, DBP: diastolic blood pressure, HR: heart rate, TC: total cholesterol, HDL-c: high-density lipoprotein cholesterol, LDL-c: low-density lipoprotein cholesterol, TG: triglycerides, FG: fasting glucose, HBA1c: glycated haemoglobin, BC: blood creatinine, UC: urine creatinine, ACR: albumin-to-creatinine ratio; eGFR: glomerular filtration rate; FMD: flow-mediated dilation.

**Table 2:** Relationship between HIV, ART and cardiovascular risk factors.

		HIV^−^ (%)	HIV^+^ART^−^ (%)	HIV^+^ART^+^ (%)	Total (%)	χ^2^	*p*-Value
BP	Normal	146 (86.4)	142 (87.1)	118 (81.5%)	406 (85.1)	0.342	0.843
	HT	23 (13.6)	21 (12.9)	27 (18.5)	71 (14.9)		
WHR	Lean	117 (68.2)	94 (57.7)	99 (67.8)	310 (64.6)	18.77	<0.001
	Obese	54 (31.8)	69 (42.3)	47 (32.2)	170 (35.4)		
TC	Normal	106 (92.2)	121(92.1)	82 (87.2)	309 (91.2)	2.535	0.282
	High	9 (7.8)	9 (6.9)	12 (12.8)	30 (8.8)		
HDL-c	Normal	160 (93.0)	147 (90.2)	141 (96.6)	348 (93.1)	14.74	0.022
	Low	12 (7.0)	16 (9.8)	5 (3.4)	33 (6.9)		
LDL-c	Normal	166 (96.5)	156 (95.7)	142 (97.3)	464 (96.5)	11.69	0.069
	High	6 (3.5)	7 (4.3)	4 (2.7)	17 (3.5)		
TG	Normal	162 (94.2)	151 (82.6)	131 (89.7)	444 (92.3)	0.089	0.352
	High	10 (5.8)	12 (7.4)	15 (10.3)	37 (7.7)		
AU	Normal	170 (99.4)	155 (95.1)	138 (94.5)	464 (96.5)	7.707	0.021
	High	1 (0.6)	8 (4.9)	8 (5.5)	17 (3.5)		

Chi-square test was used for analysis. HIV^+^ART^−^: HIV-positive participants not on ART, HIV^+^ART^+^: HIV-positive participants on ART. HIV: human immunodeficiency virus, HIV^+^: HIV-positive subjects, HIV^−^: HIV-negative subjects, WHR: waist-to-hip ratio, BP: blood pressure, HT: hypertension, HDL-c: high-density lipoprotein cholesterol, LDL-c: low-density lipoprotein cholesterol, TC: total cholesterol, TG: triglycerides; AU: albuminuria, χ^2^: chi-square.

**Table 3:** Effect of ART and hypertension on cardiovascular risk factors.

	HIV^−^		HIV^+^ART^−^		HIV^+^ART^+^		*p*-Value
Group (n)	NT (146)	HT (23)	NT (142)	HT (21)	NT (118)	HT (27)	
BMI (kg/m^2^)	26.2 ± 6.2	25.4 ± 4.5	22.3 ± 2.8	23.3 ± 3.2	23.9 ± 4.1	26.3 ± 6.3	0.649
WHC	0.8 ± 0.1	0.8 ± 0.1	0.8 ± 0.2	0.7 ± 0.2	0.8 ± 0.1	0.9 ± 0.1	0.566
TC (mmol/L)	3.9 ± 0.9	3.6 ± 1.6	4.2 ± 1.2	3.9 ± 0.6	4.1 ± 0.9	12.7 ± 19.3	0.006
HDL-c (mmol/L)	1.3 ± 0.4	1.2 ± 0.4	1.2 ± 0.4	1.4 ± 0.5	1.4 ± 0.3±	1.4 ± 0.6	0.652
LDL-c (mmol/L)	2.1 ± 0.7	1.3 ± 1.7	2.5 ± 0.9	2.1 ± 0.8	2.1 ± 0.8	10.5 ± 19.5	0.005
TG (mmol/L)	1.0 ± 0.4	1.9 ± 0.9	1.2 ± 0.6	1.1 ± 0.4	1.2 ± 0.4	1.7 ± 0.8	0.014
FBG (mmol/L)	4.9 ± 0.6	4.9 ± 0.3	4.9 ± 0.6	5.1 ± 0.9	5.1 ± 0.5	5.3 ± 0.4	0.904
HBA1c (%)	5.3 ± 0.4	5.4 ± 0.4	5.4 ± 0.2	5.0 ± 0.4	5.6 ± 0.4	5.6 ± 0.4	0.096
BC (μmol/L)	68.6 ± 11.6	76.2 ± 10.0	73.0 ± 19.9	72.4 ± 10.2	74.0 ± 13.6	64.2 ± 8.8	0.176
UC (mmol/L)	13.8 ± 6.9	15.9 ± 6.5	15.8 ± 8.5	12.1 ± 6.8	13.5 ± 6.4	14.0 ± 2.8	0.448
Albumin (mg/L)	3.8 ± 11.7	7.7 ± 8.7	1.9 ± 2.2	0.9 ± 0.6	2.6 ± 3.3	8.1 ± 8.9	0.539
ACR (mg/mmol)	0.3 ± 0.9	0.5 ± 0.7	0.1 ± 0.2	0.1 ± 0.1	0.3 ± 0.5	0.6 ± 0.7	0.789
FMD (%)	12.6 ± 10.3	11.2 ± 6.7	13.5 ± 9.8	14.7 ± 9.2	11.4 ± 19.1	11.3 ± 11.7	0.939

Multivariate analysis of variance (MANOVA) was used for analysis. Results are expressed as mean ± SD. SD: standard deviation, NT: normotension, HT: hypertension, HIV^+^ART^−^: HIV-positive participants not on ART, HIV^+^ART^+^: HIV-positive participants on ART. HIV: human immunodeficiency virus, HIV^+^: HIV-positive subjects, HIV^−^: HIV-negative subjects, BMI: body mass index, HC: hip circumference, WC: waist circumference, WHR: waist-to-hip ratio, SBP: systolic blood pressure, DBP: diastolic blood pressure, HR: heart rate, TC: total cholesterol, HDL-c: high-density lipoprotein cholesterol, LDL-c: low-density lipoprotein cholesterol, TG: triglycerides; FG: fasting glucose, HBA1c: glycated haemoglobin, BC: blood creatinine, UC: urinary creatinine, ACR: albumin-to-creatinine ratio; FMD: flow-mediated dilation.

**Table 4:** Relationship between ART, endothelial function and cardiovascular risk factors.

	HIV^−^				HIV^+^ART^−^				HIV^+^ART^+^				
FMD (*n*)	1st (41)	2nd (38)	3rd (38)	4th (35)	1st (25)	2nd (36)	3rd (41)	4th (42)	1st (37)	2nd (37)	3rd (31)	4th (27)	*p*-Value
BMI (kg/m^2^)	26.8 ± 6.2	24.9 ± 4.5	26.9 ± 7.1	26.3 ± 6.9	22.5 ± 3.2	21.6 ± 2.2	21.9 ± 2.9	23.5 ± 2.8	24.1 ± 3.7	26.4 ± 9.1	26.6 ± 4.3	23.3 ± 1.2	0.886
WHR	0.86 ± 0.2	0.79 ± 0.19	0.81 ± 0.2	0.79 ± 0.1	0.82 ± 0.1	0.86 ± 0.1	0.81 ± 0.3	0.73 ± 0.3	0.8 ± 0.1	0.91 ± 0.1	0.92 ± 0.1	0.81 ± 0.1	0.763
TC (mmol/L)	3.8 ± 1.2	3.7 ± 1.1	4.4 ± 0.8	3.8 ± 0.7	3.7 ± 1.1	4.3 ± 0.5	3.9 ± 1.1	4.7 ± 1.3	4.1 ± 1.0	16.4 ± 23.8	3.8 ± 1.3	4.8 ± 1.6	0.007
HDL-c (mmol/L)	1.3 ± 0.5	1.3 ± 0.4	1.3 ± 0.2	1.4 ± 0.5	1.2 ± 0.7	1.3 ± 0.1	1.1 ± 0.4	1.4 ± 0.3	1.4 ± 0.4	1.4 ± 0.6	1.5 ± 0.4	1.3 ± 0.3	0.938
LDL-c (mmol/L)	1.9 ± 0.7	2.0 ± 1.2	2.6 ± 0.7	1.9 ± 0.7	1.9 ± 0.7	2.5 ± 0.8	2.3 ± 0.9	2.8 ± 1.3	2.1 ± 0.9	14.1 ± 24.09	1.9 ± 0.9	3.1 ± 1.4	0.011
TG (mmol/L)	1.4 ± 0.6	1.0 ± 0.5	0.9 ± 0.6	1.0 ± 0.4	1.2 ± 0.6	1.2 ± 0.4	1.2 ± 0.7	1.1 ± 0.4	1.5 ± 0.7	1.7 ± 0.3	0.9 ± 0.0	0.9 ± 0.3	0.555

Multivariate analysis of variance (MANOVA) was used for analysis. Results are expressed as mean ± SD. SD: standard deviation, 1st quartile: <25th percentile of FMD (<6.45%), 2nd quartile: 25th to <50th percentile of FMD (6.45 to <10.48%), 3rd quartile: 50 to <75th percentile of FMD: (10.48 to <16.72%), 4th quartile: >75 to 100th percentile of FMD (≥16.72%), HIV^+^ART^−^: HIV-positive participants not on ART, HIV^+^ART^+^: HIV-positive participants on ART. HIV: human immunodeficiency virus, HIV^+^: HIV-positive subjects, HIV^−^: HIV-negative subjects, BMI: body mass index, WHR: waist-to-hip ratio, TC: total cholesterol, HDL-c: high-density lipoprotein cholesterol, LDL-c: low-density lipoprotein cholesterol, TG: triglycerides.
